# Commentary: The landscape of transcription errors in eukaryotic cells

**DOI:** 10.3389/fgene.2017.00219

**Published:** 2017-12-14

**Authors:** Bert M. Verheijen, Fred W. van Leeuwen

**Affiliations:** ^1^Laboratory of Experimental Neurology, University Medical Center Utrecht, Utrecht University, Utrecht, Netherlands; ^2^Department of Neuroscience, Maastricht University, Maastricht, Netherlands

**Keywords:** RNA, transcriptional infidelity, epimutation, molecular misreading, proteotoxicity, cellular quality control, aging, disease

The flow of genetic information within biological systems, as described by the central dogma of molecular biology (Crick, 1970), lies at the heart of life. Dedicated molecular machinery has evolved to carry out the process of replicating and converting genetic information with great accuracy. However, due to the inherently noisy nature of biological systems it is inevitable that errors can arise at any stage of life during this process (Drummond and Wilke, 2009; Tawfik, 2010). Such errors may exert significant effects on the functioning of cells—with detrimental outcomes. Propagation of errors in genetic information transfer has been surmised as one of the main molecular causes of cellular aging (Szilard, 1959; Orgel, 1963, 1970). Yet, little is known about the origins and consequences of erroneous gene expression in cells.

Transcriptional infidelity, i.e., the inaccurate conversion of DNA to RNA, constitutes one class of information transmission errors. By expressing an error-prone version of RNA polymerase II (RNAPII), increased rates of transcriptional promiscuity have been demonstrated to induce proteotoxicity and reduce cellular longevity in yeast (Vermulst et al., 2015). The error rate of transcription increases with age, which contributes to the decline in proteostasis seen in aging cells (Figure 1A).

**Figure 1:**
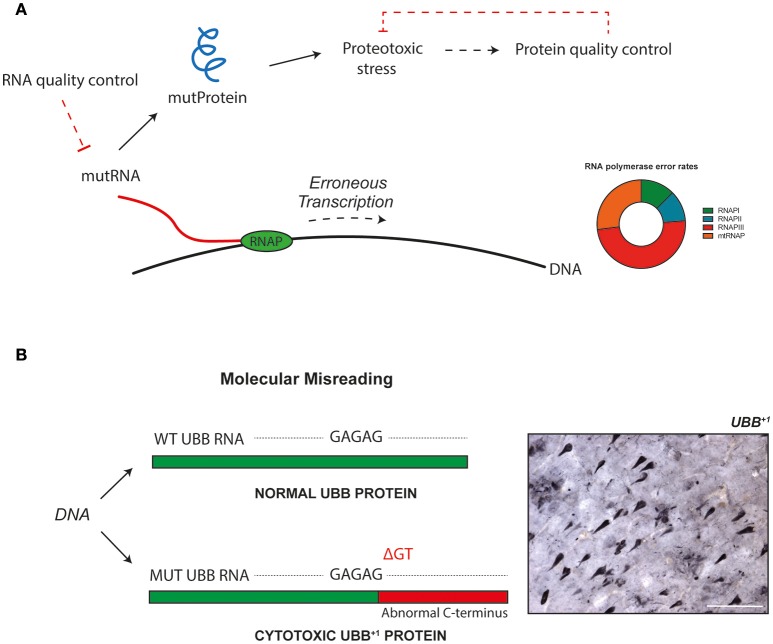
Erroneous gene transcription can bring about abnormal proteins with cytotoxic properties. These molecules impact cellular function, and may be implicated in aging and disease. Dirty transcripts yield toxic proteins. **(A)** Transcriptional misreading, i.e., the inaccurate conversion of DNA to RNA by RNA polymerase (RNAP), results in mutant transcripts that can be translated into abnormal proteins. These mutant proteins induce proteotoxic stress in cells and impact cellular physiology. Cells use several mechanisms for quality control of gene expression, to prevent abnormal molecules from being generated and to accumulate. In addition to the proofreading abilities of polymerase enzymes, specific mechanisms exist to control fidelity beyond synthesis. For example, mRNA surveillance pathways ensure mRNA quality (Doma and Parker, 2007), and abnormal proteins can be degraded by an assembly of chaperons, the ubiquitin-proteasome system and autophagy (Goldberg, 2003). It has been suggested that mutations in synthesis machinery could result in a positive feedback loop, corresponding to an error catastrophe (Martin and Bressler, 2000). Impaired quality control mechanisms, perhaps the result of errors during synthesis of their constituents, could also contribute to such a phenomenon. **(B)** Molecular misreading has been found to introduce sequence differences into nascent transcripts and generates frame-shifted proteins with cytotoxic properties. Ubiquitin-B^+1^ (UBB^+1^) is an example of such a protein and accumulates in the neuropathological hallmarks of Alzheimer's disease. It has been shown that when the levels of proteotoxic stress induced by UBB^+1^ surpass cellular redundancy, this will lead to pathogenicity. The insert shows a section from an Alzheimer's disease patient brain, containing several UBB^+1^-positive structures. Bar = 100 μm.

The introduction of optimized RNA sequencing assays allows for accurate measurements of erroneous transcription rates in cells (Reid-Bayliss and Loeb, 2017). In a recent study published in *Science Advances*, Gout et al. introduce an optimized “circle-sequencing (CircSeq) assay” and provide the first comprehensive analysis of transcription errors in eukaryotic cells (Gout et al., 2017). They demonstrate that transcription errors occur across the entire genome of the budding yeast *Saccharomyces cerevisiae* and that these errors can affect cellular function. This potentially has widespread implications for our understanding of cell physiology, aging, and disease.

The sequencing approach was adapted from the field of virology, where it is used to sequence RNA virus populations (Acevedo and Andino, 2014; Acevedo et al., 2014). Gout et al. show that the yeast transcriptome contains on average 4.0 errors per million base pairs, which implies that RNA mutation rate is over a 100-fold higher than DNA mutation rate (Lynch et al., 2016). Importantly, the adopted strategy enables them to absolutely know *which* errors come about *where* and *when*. The error spectrum of transcription reveals that errors are not distributed equally over the transcriptome, with different types of polymerases, transcribing different types of RNA, generating different amounts of errors (Figure 1A). Additionally, the measurements demonstrate that there is a limit to the capacity of non-sense mediated RNA decay to recognize erroneous transcripts.

The researchers also explore the physiological effects of transcriptional infidelity using multiple approaches. In line with previous findings, increased transcriptional error rates induce proteotoxic stress and reduce cell growth and longevity. Other biological changes include perturbation of metabolic processes, which might resemble the metabolic changes seen in senescent and diseased cells.

The widespread existence of RNA mutations may have far-reaching implications. Identification of transcription errors and thorough analysis of their phenotypic effects could lead to novel insights into aging- and disease-related loss of cellular homeostasis. Mutator phenotype in cancer is a prominent example of how an increase in error frequency contributes to cellular dysfunction (Loeb, 2016). Previous work has revealed a specific type of mistranscription to take place in human cells. Through a mechanism dubbed “molecular misreading,” which introduces dinucleotide deletions (e.g., ΔGA, ΔGU) into repeated dinucleotide runs of sequences (e.g., GAGAG) of RNA, aberrant proteins can be produced (van Leeuwen et al., 1998a,b; Bridges, 1999; Figure 1B). Although the exact causes of these errors are poorly understood, one potential mechanism for the generation of mutant transcripts is the slippage, or “stuttering,” of RNA polymerases on certain repeat motifs in DNA. Molecular misreading-derived mutant proteins have been found to accumulate in the pathological hallmarks of a number of human diseases, including Alzheimer's disease (van Leeuwen et al., 1998b). Expression of these abnormal proteins in both *in vitro* and *in vivo* experimental model systems has shown that they are potent cytotoxic agents. For example, one of the anomalous proteins, a frame-shift mutant of ubiquitin-B (UBB^+1^), induces toxicity through interfering with protein quality control systems and mitochondrial function (Krutauz et al., 2014; Braun et al., 2015; Figure 1B).

It is important to point out that transcription errors may not exclusively result in “dirty RNAs” with deleterious effects, but could also give rise to products with beneficial properties under certain conditions (Drummond and Wilke, 2009; Tawfik, 2010). The generation of alternative transcripts that increase cellular fitness might actually explain why biological systems would have some degree of error-dependent transcriptional plasticity or act on a on a “principle of limited sloppiness.” Cells are equipped with factors for the programmed generation of RNA-DNA differences via RNA editing; transcriptional mutations might contribute to normal cellular function. Furthermore, these mutations have been hypothesized to contribute to the evolution of viruses and bacteria, and development of drug resistance (Vignuzzi et al., 2006; Morreall et al., 2013). Notably, transcription errors, although transient in nature, may result in heritable changes in cellular phenotypes, as has been indicated in studies utilizing the *lac* operon in bacteria (Gordon et al., 2009, 2013; Gamba and Zenkin, 2017). Additionally, it should be stressed that RNAs are not just templates for protein synthesis, but can interact with proteins, DNA and other RNAs, and can have catalyzing properties themselves. The notion that RNAs could switch to other functional states by the introduction of error-induced sequence differences should be explored further.

The hereinbefore-mentioned study by Gout et al. on transcriptional infidelity in yeast contributes to our understanding of the diversity of the transcriptional landscape. We anticipate that more non-genetic mutations that occur during transcription will be identified in future studies. This could contribute to a better understanding of aging and disease and may also result in novel therapeutic targets. The approach devised by Gout et al. provides an excellent experimental framework for investigating transcription error rates in other cell types (e.g., neurons, myocytes) and to examine the effects of aging, disease, DNA damage, and specific genes, RNAs and proteins on transcriptional fidelity.

## Author contributions

All authors listed have made a substantial, direct, and intellectual contribution to the work, and approved it for publication.

### Conflict of interest statement

The authors declare that the research was conducted in the absence of any commercial or financial relationships that could be construed as a potential conflict of interest.
